# Primary cardiac lymphoma with central nervous system relapse

**DOI:** 10.1002/ccr3.1094

**Published:** 2017-07-20

**Authors:** Cleo R. van Rooijen, Asbjorn M. Scholtens, C. Niels de Jong, Colette E. Saraber, Niels W. C. J. van de Donk

**Affiliations:** ^1^ Department of Hematology VU University Medical Center Amsterdam The Netherlands; ^2^ Department of Radiology University Medical Center Utrecht Utrecht The Netherlands; ^3^ Department of Hematology Erasmus Medical Center Rotterdam The Netherlands; ^4^ Department of Cardiology Tergooi Hospital Hilversum The Netherlands

**Keywords:** Cardiac imaging, CNS recurrence, primary cardiac lymphoma

## Abstract

Primary cardiac lymphoma (PCL), a rare disease, often presents with symptoms resembling other cardiac diseases. The correct diagnosis is crucial, as cardiac lymphoma can be cured with immuno‐chemotherapy. PCL has a high risk of central nervous system recurrence (CNS); therefore, screening for CNS involvement and even prophylaxis may be necessary.

## Case Reports

**Figure 1:**
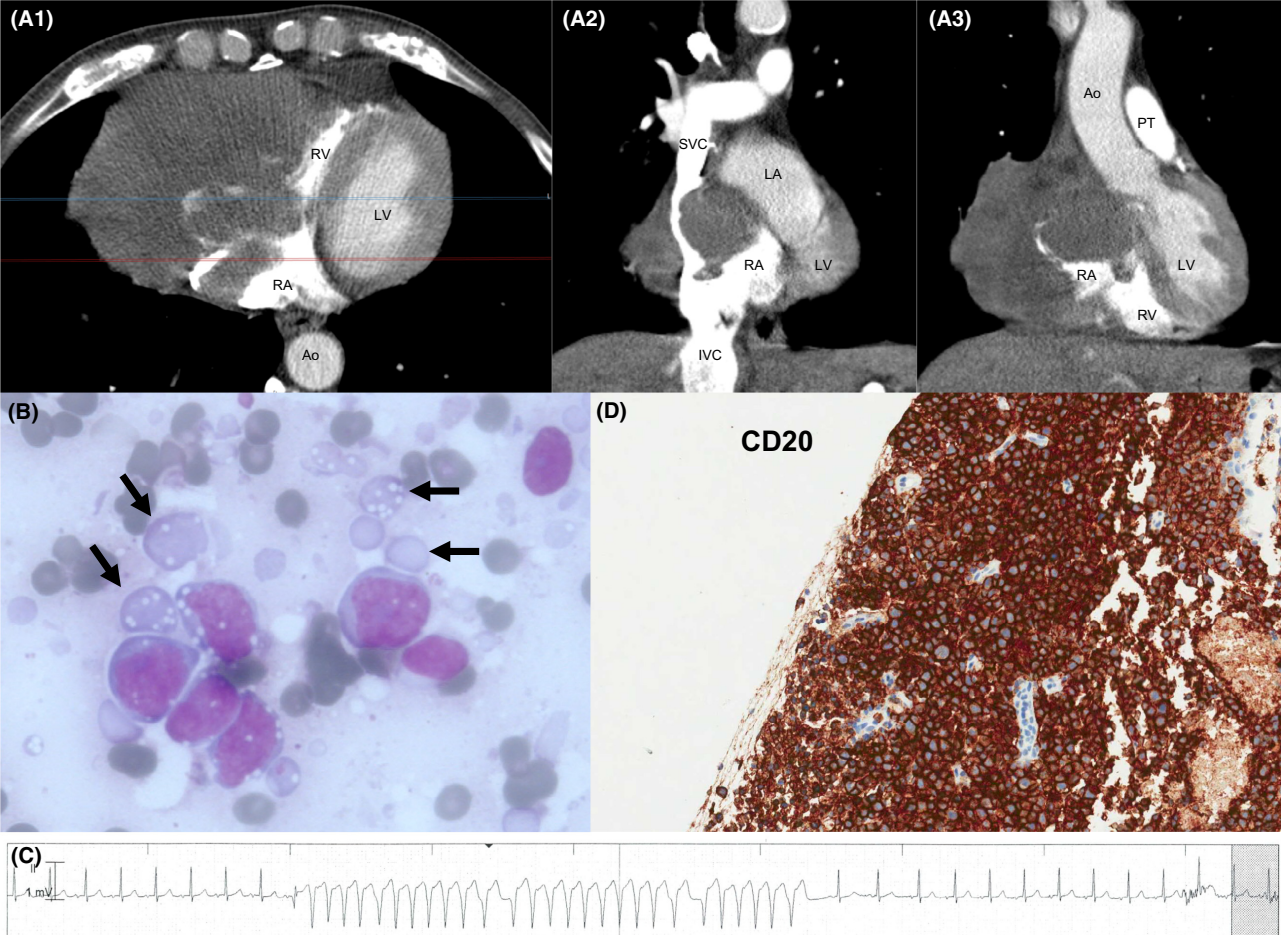
CT, cytology, histology, and ECG results from case A. (A) Contrast‐enhanced axial CT scan imaging demonstrating a large irregular mass with infiltration into the right atrial wall and right ventricular myocardium: Panel 1 is an axial view; panel 2 is a coronal view at the level of the red line in A1; panel 3 is a coronal view at the level of the blue line in A1. (B) Lymph node smear showing large blast‐like cells with prominent nucleoli, as well as various lymphoglandular bodies. (C) High power magnification showing positive staining of the tumor cells for CD20. (D) Ventricular tachycardia with normalization of the QRS complex after conversion to sinus rhythm. RA, right atrium; LA, left atrium; RV, right ventricle; LV, left ventricle; Ao, aorta; IVC, inferior vena cava; IVS, superior vena cava; PT, pulmonary trunk.

Patient A, a 69‐year‐old man with negative medical history, presented with progressive swelling of his face and both arms since 4 weeks. Upon examination we noticed edema of the face, neck, and upper extremities. Laboratory analysis revealed an elevated LDH of 704 U/L (reference range <250 U/L), mild anemia, and normal leukocyte count with normal differentiation. A contrast‐enhanced computed tomography scan of the chest demonstrated a myocardial mass, measuring 10 × 11 × 10 cm, that infiltrated the right atrium and right ventricle and caused compression of the superior vena cava (Fig. 1A). Additional imaging studies demonstrated no other sites of disease. CT‐guided biopsy was performed instantly. The same day, cytology showed large blast‐like cells with prominent nucleoli and also lymphoglandular bodies (cytoplasmic fragments of lymphocytes, frequently observed in association with lymphoid malignancies), favoring a diagnosis of large‐cell lymphoma (Fig. 1B). Therefore, upon obtaining these cytology results, prednisone was started in combination with rasburicase as tumor‐lysis prophyaxis, while he received continuous monitoring of cardiac rhythm. Two days later he experienced an episode of nonsustained ventricular tachycardia with spontaneous conversion to sinus rhythm (Fig. 1C). He also experienced episodes of atrial fibrillation and atrial tachycardia. After start of metoprolol, he maintained sinus rhythm. A few days later, histologic examination confirmed the diagnosis of diffuse large B‐cell lymphoma (DLBCL), with tumor cells positive for CD20, CD79a, CD10, BCL2, and BCL6, with a high Ki‐67 proliferative index (~80%) (Fig. 1D). As there was no bone marrow localization, it was a stage IE primary cardiac lymphoma. FISH analysis showed the absence of *BCL2*,* BCL6*, or *MYC* translocations. HIV status was negative. He was treated with the combination of rituximab, cyclophosphamide, etoposide, vincristine, and prednisone (R‐CEOP). The anthracycline doxorubicin was omitted and replaced with etoposide, as there was a reduced left ventricular ejection fraction of 38%. After 6 cycles of R‐CEOP and two additional infusions of rituximab he obtained a partial response, with improvement of the left ventricular ejection fraction (51%). Because of poor performance status, no additional treatment was given. Unfortunately, 5 months after completion of therapy he developed apraxia and dysphasia resulting from multiple intracerebral lymphoma localizations. He received four cycles of high‐dose methotrexate (3 g/m^2^) and achieved a complete response. However, 5 weeks after his last course, the dysphasia recurred and magnetic resonance imaging (MRI) showed again a lesion in the left temporoparietal area. He received whole‐brain radiotherapy, after which his neurological symptoms improved. Only 2 months later his cerebral lymphoma relapsed and he ultimately died from progressive CNS disease.

**Figure 2:**
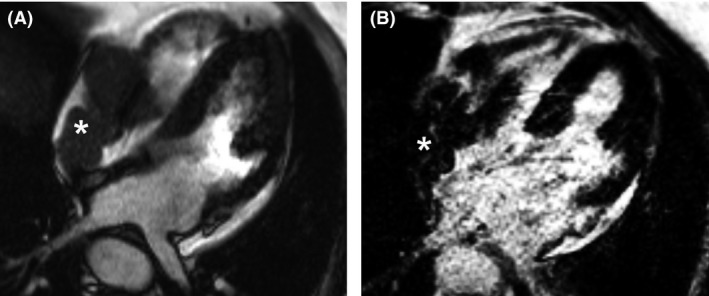
Cardiac MRI (four‐chamber view) from patient B. (A) Still frame from cine series showing the tumor (*) expanding from the right atrium toward the tricuspid valve and the right ventricle, and partly obstructing the tricuspid valve. (B) Late gadolinium enhancement showing partial washout in the mass (*), consistent with a malignant tumor.

Patient B, a 75‐year‐old man was admitted to the emergency department because of recurrent syncope in the last 2 months. His medical history included hypertension, paroxysmal atrial fibrillation, left ventricular hypertrophy, and angina pectoris. Laboratory evaluation and echocardiography showed no abnormalities. His electrocardiogram revealed sinus bradycardia with a left heart axis and a first degree AV block (PQ duration: 380 ms), as well as a right bundle branch block (QRS duration: 146 ms). A pacemaker was implanted because of this trifascicular block. Postimplantation, a cardiac MRI was performed as part of a clinical trial (Fig. 2). Unexpectedly, this MRI showed an endocardial tumor in the right atrium and right ventricle. The tumor also involved the tricuspid valve, thus restricting blood flow from the right atrium to the right ventricle. The patient's syncope was probably caused by this obstruction of blood flow, but arrhythmias could also have played a role. No other tumor localizations were found on FDG‐PET/CT. He subsequently underwent intracardiac echocardiography‐guided biopsy of the tumor. Cytologic examination showed blast‐like cells with nucleoli. Immunohistochemical staining was positive for CD20 and PAX5 confirming the diagnosis of DLBCL. There was not enough tissue for FISH analysis. A staging bone marrow biopsy showed no localization of lymphoma; therefore, he had a stage IE primary cardiac lymphoma. HIV status was negative. The patient had a good left ventricular ejection fraction and was treated with six cycles of R‐CHOP (rituximab, cyclophosphamide, doxorubicin, vincristine, and prednisone) followed by two cycles of rituximab alone. He achieved a complete response.

Two years after the initial presentation he experienced rapidly progressive cognitive decline as a result of central nervous system (CNS) relapse with multifocal parenchymatous lesions on MRI. He quickly recovered after starting dexamethasone and subsequently received high‐dose methotrexate (3 g/m^2^). Although initially responding to therapy, he died 2 months after initiation of chemotherapy due to progressive disease.

## Discussion

Cardiac involvement in lymphoma is very rare. This includes primary cardiac lymphoma (PCL), which is defined as lymphoma involving only the heart and/or pericardium without dissemination 1, 2, and secondary involvement of the heart by lymphoma which is more common and often involves late stage lymphoma and is frequently only diagnosed during postmortem examinations 3, 4. Although any tumor can metastasize to the heart, the most common tumors include carcinomas of the lung and breast, melanoma, lymphoma, and leukemia 3. Approximately 75% of the primary cardiac masses are benign. PCL accounts for approximately 1–2% of all primary cardiac tumors. Diffuse large B‐cell lymphoma is the most common lymphoma subtype, and the right side of the heart is more frequently involved, as opposed to the left‐side predominance of benign myxomatous tumors of the heart 5, 6. The clinical presentation is variable and depends on cardiac site of involvement (endocardial/intracavitary, myocardial, or epicardial/pericardial), size, growth rate, and degree of invasion 6. Frequently observed are valvular dysfunction, heart failure due to myocardial infiltration, superior vena cava syndrome, tumor embolization, conduction defects such as atrioventricular block, and arrhythmias (mainly atrial but also ventricular) 5, 7. Furthermore, pericardial infiltration may result in pericardial effusion or tamponade. Also cardiac ischemia resulting from lymphomatous deposits has been described 8. Sudden death may occur as a result of ventricular rupture, acute myocardial infarction, or ventricular arrythmias 9, 10, 11.

There is a paucity of literature on the treatment of PCL 5. However, the available data suggest that lymphoma with cardiac involvement should be treated with combination (immuno)chemotherapy. Autologous stem cell transplantation can be used in younger patients with primary refractory lymphoma, in case of relapsed disease, or as consolidation as part of first‐line therapy in case of certain high‐risk lymphoma types such as certain T‐cell non‐Hodgkin lymphomas. Radiotherapy can be considered in elderly and frail patients not achieving complete response to (immuno)chemotherapy. Furthermore, in the presence of bulky disease, radiotherapy can be given to consolidate systemic therapy to further improve local control. However, in long‐term survivors radiotherapy carries the risk of radiation‐induced heart complications such as cardiomyopathy, diastolic dysfunction, and coronary artery disease 12. Importantly, clinicians should be aware that cardiac lymphoma may be accompanied with life‐threatening rhythm disturbances (as in these cases), as well as cardiac wall perforation which can occur at diagnosis and in response to treatment. These possible complications should be discussed with the patient, and chemotherapy should be started in combination with cardiac monitoring. Overall survival of PCL is poor compared to other types of extranodal lymphomas such as primary gastric, breast, or testicular lymphoma 5, 13, 14, 15.

Our two patients developed a CNS relapse at 6 months and 2 years from initial diagnosis. We identified six additional PCL cases in the literature, who also developed isolated CNS recurrence of the lymphoma 16, 17, 18, 19. CNS disease typically developed within a few months after initial presentation, raising the question whether occult CNS localization was already present at the time of diagnosis. Similar to our two patients, all patients with CNS relapse described in the literature were treated with methotrexate‐based chemotherapy with or without radiotherapy. Younger patients also received consolidation with high‐dose chemotherapy and autologous stem cell rescue 16. Most patients described in these case reports achieved (near) complete remission. However, information on duration of response and survival was not available in most cases, because of short follow‐up. Although our patients showed an initial response to chemotherapy, both died due to progressive disease early after initiation of chemotherapy.

Altogether, our data suggest that PCL patients are at an increased risk of developing CNS relapse. Based on available data we recommend screening at the time of diagnosis for occult CNS involvement with MRI of the brain and CSF analysis, and subsequent treatment of patients with subclinical CNS disease. Alternatively, CNS prophylaxis can be considered in all patients with PCL. However, it is currently unknown whether CNS prophylaxis at diagnosis will prevent development of CNS relapse. Moreover, the debate on the optimal route of CNS prophylaxis (intrathecal versus systemic) is still ongoing.

In conclusion, PCL is a rare entity that can present with symptoms caused by heart failure or arrhythmia. Furthermore, PCL is probably associated with a high risk of CNS relapse, and therefore, in our opinion, either screening for CNS involvement or prophylactic therapy should be considered in all patients presenting with PCL.

## Conflicts of Interest

None declared.

## Authorship

CRR: wrote parts of the manuscript and reviewed the manuscript. AMS: provided figures and legends, reviewed the manuscript. CNJ: wrote parts of the manuscript and reviewed the manuscript. CES: provided figures and legends, reviewed the manuscript. NWCJD: wrote parts of the manuscript and reviewed the manuscript.

## References

[1] Mato, A. R. , A. K. Morgans , M. R. Roullet , A. Bagg , E. Glatstein , H. I. Litt , et al. 2007 Primary cardiac lymphoma: utility of multimodality imaging in diagnosis and management. Cancer Biol. Ther. 6:1867–1870. 18075298 10.4161/cbt.6.12.5166

[2] Nonami, A. , K. Takenaka , K. Kamezaki , T. Miyamoto , N. Harada , K. Nagafuji , et al. 2007 Successful treatment of primary cardiac lymphoma by rituximab‐CHOP and high‐dose chemotherapy with autologous peripheral blood stem cell transplantation. Int. J. Hematol. 85:264–266. 17483065 10.1532/IJH97.06197

[3] Bussani, R. , F. De‐Giorgio , A. Abbate , and F. Silvestri . 2007 Cardiac metastases. J. Clin. Pathol. 60:27–34. 17098886 10.1136/jcp.2005.035105 PMC1860601

[4] Chinen, K. , and T. Izumo . 2005 Cardiac involvement by malignant lymphoma: a clinicopathologic study of 25 autopsy cases based on the WHO classification. Ann. Hematol. 84:498–505. 15782345 10.1007/s00277-005-1009-5

[5] Petrich, A. , S. I. Cho , and H. Billett . 2011 Primary cardiac lymphoma: an analysis of presentation, treatment, and outcome patterns. Cancer 117:581–589. 20922788 10.1002/cncr.25444

[6] Grebenc, M. L. , M. L. Rosado de Christenson , A. P. Burke , C. E. Green , and J. R. Galvin . 2000 Primary cardiac and pericardial neoplasms: radiologic‐pathologic correlation. Radiographics 20:1073–1103. 10903697 10.1148/radiographics.20.4.g00jl081073

[7] Gowda, R. M. , and I. A. Khan . 2003 Clinical perspectives of primary cardiac lymphoma. Angiology 54:599–604. 14565636 10.1177/000331970305400510

[8] O'Mahony, D. , R. L. Peikarz , W. P. Bandettini , A. E. Arai , W. H. Wilson , and S. E. Bates . 2008 Cardiac involvement with lymphoma: a review of the literature. Clin. Lymphoma Myeloma 8:249–252. 18765314 10.3816/CLM.2008.n.034 PMC2998057

[9] Lauten, M. , S. Vieth , C. Hart , W. Wössmann , B. Tröger , C. Härtel et al. 2014 Cardiac anaplastic large cell lymphoma in an 8‐year old boy. Leuk. Res. Rep. 3:36–37. 24918065 10.1016/j.lrr.2014.05.001 PMC4050286

[10] Abdullah, H. N. , and W. K. Nowalid . 2014 Infiltrative cardiac lymphoma with tricuspid valve involvement in a young man. World J. Cardiol. 6:77–80. 24575174 10.4330/wjc.v6.i2.77 PMC3935062

[11] Molajo, A. O. , L. McWilliam , C. Ward , and A. Rahman . 1987 Cardiac lymphoma: an unusual case of myocardial perforation–clinical, echocardiographic, haemodynamic and pathological features. Eur. Heart J. 8:549–552. 3609049 10.1093/oxfordjournals.eurheartj.a062317

[12] Curigliano, G. , D. Cardinale , T. Suter , G. Plataniotis , E. de Azambuja , M. T. Sandri , et al. 2012 Cardiovascular toxicity induced by chemotherapy, targeted agents and radiotherapy: ESMO Clinical Practice Guidelines. Ann. Oncol. 23(Suppl 7):vii155–vii166. 22997448 10.1093/annonc/mds293

[13] Cortelazzo, S. , A. Rossi , F. Roggero , E. Oldani , E. Zucca , C. Tondini et al. 1999 Stage‐modified international prognostic index effectively predicts clinical outcome of localized primary gastric diffuse large B‐cell lymphoma. International Extranodal Lymphoma Study Group (IELSG). Ann. Oncol. 10:1433–1440. 10643533 10.1023/a:1008351427601

[14] Ryan, G. , G. Martinelli , M. Kuper‐Hommel , R. Tsang , G. Pruneri , K. Yuen . et al. 2008 Primary diffuse large B‐cell lymphoma of the breast: prognostic factors and outcomes of a study by the International Extranodal Lymphoma Study Group. Ann. Oncol. 19:233–241. 17932394 10.1093/annonc/mdm471

[15] Zucca, E. , A. Conconi , T. I. Mughal , A. H. Sarris , J. F. Seymour , U. Vitolo et al. 2003 Patterns of outcome and prognostic factors in primary large‐cell lymphoma of the testis in a survey by the International Extranodal Lymphoma Study Group. J. Clin. Oncol. 21:20–27. 12506165 10.1200/JCO.2003.11.141

[16] Jung, Y. H. , I. S. Woo , Y. J. Ko , J. H. Lee , J. W. Lim , C. W. Han et al. 2014 A case of primary cardiac lymphoma showing isolated central nervous system relapse. Clin. Lymphoma Myeloma Leuk. 14:e31–e33. 24220617 10.1016/j.clml.2013.09.003

[17] Montoro, J. , L. Mattia , P. Bertazzoni , S. Liptrott , N. Colombo , M. Civelli et al. 2014 Primary cardiac lymphoma with isolated parenchymal central nervous system relapse: report of two cases and review of the literature. Ecancermedicalscience 8:474. 25374622 10.3332/ecancer.2014.474 PMC4217537

[18] Nishizawa, M. , K. Yamashita , Y. Nakamoto , S. Kotani , T. Kondo , and A. Takaori‐Kondo 2010 Neurolymphomatosis as a manifestation of relapsed primary cardiac lymphoma. Int. J. Hematol. 92:679–680. 21072622 10.1007/s12185-010-0716-4

[19] Bulum, J. , L. Banfic , M. Strozzi , I. Aurer , and D. Jelasić 2007 Primary cardiac lymphoma presenting as atrial flutter and total heart block. Heart Vessels 22:52–54. 17285447 10.1007/s00380-006-0924-2

